# Qualitative program evaluation of social determinants of health screening and referral program

**DOI:** 10.1371/journal.pone.0242964

**Published:** 2020-12-01

**Authors:** Vivian N. Emengo, Myia S. Williams, Rachael Odusanya, Omolara T. Uwemedimo, Johanna Martinez, Renee Pekmezaris, Eun Ji Kim

**Affiliations:** 1 Donald and Barbara Zucker School of Medicine at Hofstra/Northwell, Hempstead, New York, United States of America; 2 Institute of Health Innovations and Outcomes Research, Feinstein Institutes for Medical Research, Manhasset, New York, United States of America; 3 Lewis Katz School of Medicine at Temple University, Philadelphia, Pennsylvania, United States of America; The MetroHealth System and Case Western Reserve University, UNITED STATES

## Abstract

Although the integration of social determinants of health (SDH) screening and referral programs in clinical settings has rapidly grown, the voice and experience of participants within SDH programs has not been well understood in program evaluations. To qualitatively evaluate a comprehensive SDH screening and referral program based in an academic primary care setting, we conducted a qualitative analysis of a semi-structured, focus group interview of 7 caregivers. We performed inductive coding representing emerging ideas from each transcript using focus group transcripts from families who participated in the SDH screening and referral program. A thematic model was created describing caregivers’ experiences with respect to screening, intake, and referral phases of the program. Caregivers reported satisfaction with structural and process-related components of screening, intake, and referral. They expressed a preference for trained patient navigators over physicians for screening and intake for they were perceived to have time to prioritize caregivers’ social needs. Caregivers reported disappointment with legal services screening, intake, and referral, citing lack of timely contact from the legal resource team and prematurity of provided legal resources. Overall, caregivers recommend the program, citing that the program provided social support, an environment where expression is encouraged, motivation to address their own health needs, and a convenient location. Overall, caregivers would recommend the program because they feel socially supported. The use of trained patient navigators appears to be instrumental to the successful implementation of the program in clinics, for navigators can provide caregivers with the appropriate time and personal attention they need to complete the survey and discuss their needs. Streamlining the referral process for evaluation of health-harming needs by the medical legal partnership was highlighted as an area for improvement.

## Introduction

A number of studies have documented the contribution of unmet social needs [1–3], including income [4], housing [5, 6], food [7, 8], employment, education [9], and experiences of discrimination [10]—to poor healthcare experiences, decreased healthcare access, and adverse health outcomes [4, 11–13]. In the short term, addressing unmet social needs protects children from increased risk of childhood illness, such as asthma [14], childhood obesity, anxiety, depression, and trauma [15]. Additionally, unmet social needs in childhood have been shown to have lasting effects over the course of an individual’s lifespan [11]. Poor socioeconomic conditions in childhood have been linked to increased risk of chronic diseases—including adult-onset diabetes, respiratory disease, and stomach cancer [11]—and poorer outcomes in adulthood compared to patients without socioeconomic disadvantage [11]. For the potential short- and long-term prevention benefits, addressing unmet social needs in childhood has received considerable attention [11]. Evidence of life-course influences on adult health provides a powerful rationale for policies that prioritize investment in improving the social conditions of children as a strategy for improving health and reducing health disparities across the entire life course [11].

Social determinants of health (SDH) screening and referral programs have been implemented in pediatric clinical settings over the last decade [16–19]. However, only a limited number of programs have rigorously evaluated these programs [16, 20]. Comprehensive pediatric screening and referral programs have reported some success; a randomized controlled trial of one such program demonstrated that systematically screening and referring for unmet socioeconomic needs during pediatric primary care visits resulted in an increase in use of community resources for families [20]. Overall, the majority of program evaluations have focused on metrics such as process measures, changes in social need, health impacts, financial impacts, and provider impacts but have often omitted an understanding of participants’ experiences within the program [16].

To understand the perceptions and experiences of program participants, this study provides qualitative analysis of participant feedback on the Social Health Alliance to Promote Equity (SHAPE) comprehensive SDH screening and referral program based in the Division of General Pediatrics primary care practice of Cohen Children’s Medical Center at Northwell Health. The program screens families for unmet social needs in the pediatric clinical setting and refers families to community-based resources and partner organizations, as well as a medical-legal partnership. Given the dearth of information on caregiver perceptions and experience on SDH screening and referral programs, this paper aims to leverage existing feedback from a focus group with families participating in the SHAPE Program in order to provide insight into this population’s attitudes and beliefs on these programs and guidance for areas of improvement.

## Methods

### Study population

**Fig 1:**
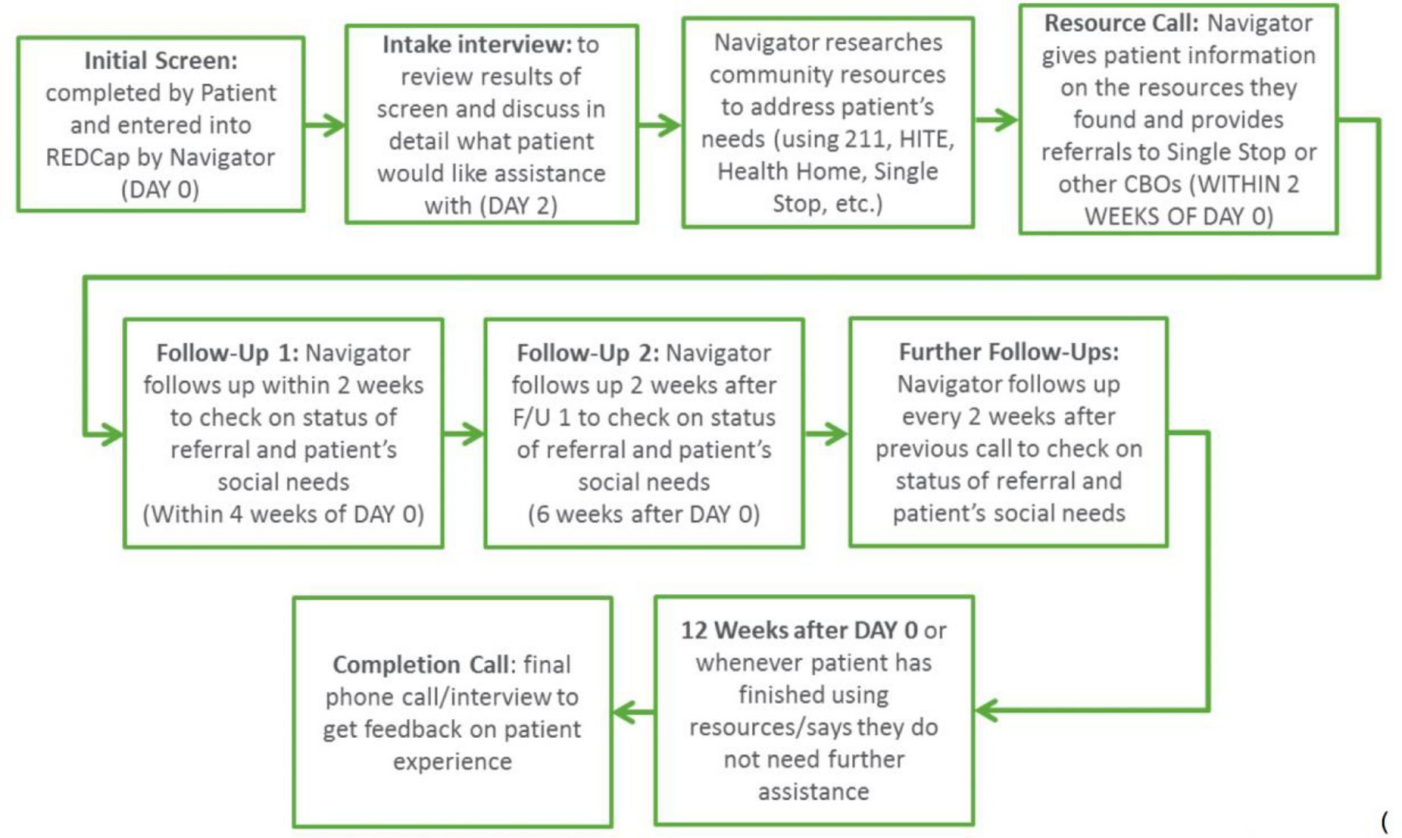
SHAPE SDH screening and referral program flowchart.

Northwell Health’s Cohen’s Children Medical Center-General Pediatrics (CCMC-GP) Practice implemented the SHAPE Screening Program [previously referred to as the Family Needs Screening Program] [19]. SDH screening occurs during pediatric well-child visits: caregivers received a 1-page socioeconomic needs survey, which is administered by a trained patient navigator. The screening tool assesses needs related to multiple domains including: housing, food, child care, transportation, insurance, education, employment, legal, immigration, health literacy, mental health, abuse, and discrimination. Families with unmet social needs as identified in the pediatric clinic setting are then referred to community health organizations and resources. During the process, the families receive follow-up phone calls from trained patient navigators every two weeks (Fig 1). The SHAPE was funded by the United Hospital Fund’s Partnerships for Early Childhood Development, Phase II grant to strengthen clinical-community partnerships.

To qualitatively evaluate the SHAPE program, the SHAPE Program Director (OTU) hosted a patient forum for families of patients who participated in the SDH screening and referral program. Preparations for the forum included assistance from Public Agenda, a group that has facilitated multiple forums for the United Hospital Fund [19]. Public Agenda created a general moderator guide that the Northwell team adapted to fit their own specific questions and needs. Recruitment for the forum occurred during regular follow-up phone calls between patient navigators and families every 2-weeks. Patients served by the pediatric ambulatory practice are ethnically and socioeconomically diverse with greater than half of patients living in immigrant families from Latin America, East Asia, South Asia, non-Hispanic Caribbean, the Middle East, and Africa; over two-thirds of patients receive health insurance coverage through Medicaid or Medicaid managed care. The majority of caregivers engaged in the SDH screening and referral program were predominantly mothers (83.2%), non-white (91.9%), high school educated (80.6%), immigrant (59.7%), and US Citizens (71.8%). Caregivers engaged in the focus group were predominantly African American and Latinx mothers with a smaller percentage of South East Asian mothers. Approximately one-third (30.9%) of all caregivers engaged in the program successfully utilized program-provided resources at the 12-week follow-up. Limited English proficiency caregivers were more likely to be lost-to-follow-up during the referral program. However, a higher proportion of the families who utilized the program-provided resources were caregivers with limited English proficiency compared to caregivers with English proficiency [21].

At the time of the family recruitment, there were 927 patients screened, 590 patients screened positive for at least one social need, and 190 had accepted assistance from patient navigators. There were 25 families that, regardless of whether or not they had completed the 12-week referral program, utilized referrals from the Northwell Medical Legal Partnership, Child Center of New York Single Stop program or the Interfaith Nutrition Network. Of the 10 families that were invited, 6 families attended—6 mothers and 1 father. Participants mainly spoke English at home (5 out of 6 participants), were from a racial/ethnic minority group (2 South Asian, 1 Black, 1 Hispanic, and 2 Declined to self-identify their race), had varied educational attainment (2 had less than high school degree, 3 had college degree or equivalent, and 1 had a graduate degree), and were born outside of the United States (3 out of 6 were born outside of the United States).

The forum time and duration were selected based on the convenience of families. Dinner was provided and two patient navigators assisted with on-site childcare. The forum was moderated by the SHAPE Program Director, Program Coordinator and patient navigators. Moderators used a guide with open ended questions and probes related to the screening program. Participants were queried about factors that influenced child health, perceptions of the screening program, and the quality of the referral process. The discussion was digitally recorded and stored on an internal server to ensure security and later transcribed. Transcripts were checked against the original recordings for accuracy. At the close of the forum, each family received a $75 Amazon gift card for their participation.

### Data analysis

To optimize credibility, transferability, and dependability of results, we utilized researcher triangulation, peer debriefing, and documented the trail of decisions made during the analysis and rationale. A thematic analysis approach was utilized as the primary methodological orientation [21]. The focus group transcript was analyzed by two authors, VE and MW, multiple times independently to ensure familiarity with the data. Both authors documented initial theoretical and reflective thoughts, as well as potential codes and themes. VE and MW focused on patterns in the data to generate a comprehensive set of codes. In order to make explicit how they perceived, examined and developed their understanding of the data and its source in the transcription, VE and MW explained their rationale for coding a particular block of text. Next, authors searched for themes after the coding process was initially completed and the codes were collated. Themes were reviewed and refined. Potential themes were discussed by qualitative researchers and revised. Disagreements were rectified by a mediator (EK). Lastly, all team members met and discussed definitions and finalization of theme names. We used the Donabedian framework [22] for quality assessment to classify themes into 3 broad themes—structure, process, and outcomes. The study was reviewed and approved by the Cohen’s Children Medical Center Institutional Review Board.

## Results

Overall, 13 sub-themes were categorized under the 3 main themes of structure, process and outcome (Table 1).

**Table 1:** Themes.

Structure	Process	Outcomes
(i) Screening and intake questions	(iv) Screening/intake duration/timing	(x) Caregiver perception of social support from the health care team
(ii) Trained patient navigators	(v) Alignment of intake phone calls with individual caregiver’s schedule	(xi) Screening program as a motivation tool for prioritizing health
(iii) Screening location and mode of communication during intake and referral.	(vi) Speed/timeliness of particular referrals	(xii) Direct and indirect determinants of child health
	(vii) Concordance of particular referrals with caregiver needs	(xiii) Recommendation
	(viii) Physician awareness of screening results	
	(ix) Opportunity areas for legal screening and referral process	

### Theme # 1: Structure

#### (i) Screening and intake questions

Overall, caregivers are satisfied with the SDH screening tool; specifically, with regard to location of survey administration (exam room/waiting room), length of screening survey (1-page), and survey questions.

"…everything she asked me, I felt it was pretty decent…like it wasn't nothing that I didn't have to share with her, that I felt was too confidential…”

#### (ii) Trained patient navigators

Caregivers reported satisfaction with the trained patient navigators administering the screening survey. When offered the option to receive surveys from their physician or a trained navigator, caregivers expressed a preference for the trained navigator. This preference appeared to be mediated by a perception that navigators have time to appropriately attend to caregiver concerns.

*“The navigators because they are trained*. *The doctors are here to do their job mainly so it's kind of hard to pour your heart to a doctor when they have a bunch of patients who probably want to do the same thing…so it's better that way*.*”* “*The patient navigator was good at making it feel not routine…”*

#### (iii) Screening location and mode of communication during intake and referral

Caregivers reported being comfortable with the structural components of intake, with regard to questions and mode of communication (via phone). When probed for improvements to the intake process, caregivers suggested providing additional modes of communication (e.g. email).

"It is effective over the phone" " I don't know if there is an email option"

### Theme #2: Process

#### (iv) Screening and intake duration and timing

Caregivers prefer to receive the screening survey while waiting for the physician visit, noting that this makes good use of waiting time.

“…it was something for me to do while I waited for the doctor”

#### (v) Alignment of intake phone calls with individual caregivers’ schedules

When probed for improvements to the intake process, caregivers recommended that navigators align the intake phone call with caregiver’s schedules to yield more consistent responses.

“It’s all about schedules. Everybody has different lives. We need someone available at 9 a.m., some people are going to want 9 p.m… It just depends on their lives and their work schedule.”

#### (vi) Speed/timeliness of particular referrals

On average, referrals ranged between hours to 1 week after the intake phone call and caregivers generally appear satisfied with this timing. In fact, parents were impressed by speed of referrals and relevance of referrals (e.g. the INN, employment resources) to their circumstance.

*[In reference to time between screening, intake, and referral]*:

“Actually, wasn't that long…3–4 hours and then I got a phone call.” “A week.”

#### (vii) Concordance of particular referrals with caregiver needs

Generally, caregivers expressed satisfaction with the referrals they received to resources such as food pantries and employment assistance programs.

"they know everything about me, like the navigator explained everything to them and they were like we know this part and we would want to help you. It was really impressive.”

“…I like the calls I got from the two young ladies who called me. I liked that call about the job search and stuff like that. That was nice.”

#### (viii) Physician awareness of screening results

Some caregivers revealed that their physicians received the results of their screening survey during the visit and they expressed that they appreciated when the doctor is informed about the results of the screening.

“Actually, my doctor came in right after her [patient navigator] … and she was like okay…well I am going to see what is going on, to help you”

#### (ix) Opportunity areas for legal screening and referral process

Caregivers expressed disappointment with timing, speed, and relevance of legal referrals, citing that follow-up times were longer than anticipated and that some legal referrals were premature for the patient's circumstance.

“*For the part with the lawyer*. *I didn't get the follow-ups like I thought I was going to get*, *no calls or emails from the lawyer that I thought I was going to get…” “The lawyer was really no help to me because it wasn't like a resource I could use at the moment right now to help with my situation*.*”* “[Caregiver, when asked whether they were expecting something different from the referral they received] *Well*, *yeah*, *like a better resource besides a lawyer… but at the moment because I don't have a court date*, *it is not useful at now*.*”*

### Theme #3: Outcomes

#### (x) Caregiver perception of social support from the health care team

Parents reported feeling supported when someone on the health care team takes the time to ask about, listen to, and understand the difficulties they’re having.

“*it feels good to know that someone else is thinking about you as a parent*. *Because you as a parent*, *you gotta be there for your kids*, *can't be depressed or down especially when you have to take care of your kids*. *So*, *it is important that somebody else is able to see besides people who are with you every day*.*”*

In fact, caregivers actually expect such support from their health team.

“*You expect someone there to take care of us*. *Even the doctors come in*, *ask questions*, *how are you feeling*, *give us a stress form*, *how things going on*, *are you depressed and mostly what happened to me*, *my kids*, *they helped me out…”*

#### (xi) Screening program as a motivation tool for promoting health

Interestingly, caregivers reported that SDH screening motivates them to discuss, prioritize, and care for their own health.

*“*…*I just feel like every single time it tells me something because it still reminds yourself that you have to take this as seriously as you take any other health issue.”*

#### (xii) Direct and indirect determinants of child health

When asked to identify social issues that could influence the heath of their children, caregivers identified two types of factors. Here, we use the phrase “direct determinants” to describe factors that the child experiences, while “indirect determinants” describe factors that the caregiver experiences (Table 2). Pediatric caregivers expressed concern about child exposures to disease, including chronic illnesses, mental illness and socially-acquired illnesses, as well as the child’s diet. Of particular concern were illnesses acquired from daycare exposure. For example, one parent attributed her son’s recurrent infections, including at least one contraction of hand-foot-mouth disease, to daycare interactions with a chronically-infected child. Quality, or lack thereof, of daycare facilities and staff emerged as a salient theme. In addition to its role as a potential site of infectious exposure, caregivers espoused concern that daycare environments may not provide children with adequate protection from harm due to a high ratio of students to daycare staff and unqualified/ill-equipped staff. Caregivers acknowledged two different pathways in which a child’s peers influence the child’s health: 1) increased likelihood of exposure to infectious agents; 2). engagement in behaviors deemed by caregivers to be inappropriate, dangerous, or otherwise detrimental to the child’s health. Lastly, caregivers identified the following factors that indirectly affect child health: caregiver health and mental well-being, caregiver financial constraints, unemployment/underemployment, food insecurity and social support.

**Table 2:** Direct and indirect determinants of health.

Subthemes	Representative Quotes
**Direct Determinants of Health**	
Child’s current health status	“My baby is little right now and with my mom and she has a lot of health issues, so basically her health.”
	“Anxiety and depression.”
	“…My first concern was baby was born with low blood sugar, had to give him formula and formula is pretty expensive…”
	“…He got the hand-foot-mouth…”
Daycare facilities	“You are trusting somebody else to take care of your child and multiple children at once”
	“[In reference to day care] You hear a lot more of bad things about day care than you do good”
	“My son in day care with my neighbor and got him sick so many times because of my neighbor because her daughter was in day care and then she played with him and got him sick, she was constantly sick because she was in day care.”
Child’s peer group	“Their friends.”
	“Their peers.”
	“Teenagers and kids are wild. Parents must watch their child's every move and not give them freedom for things shouldn't and can't do”
**Indirect Determinants of Health**	
Caregiver health and mental well being	“Because you as a parent, you gotta be there for your kids, can't be depressed or down especially when you have to take care of your kids…So, it is important that somebody else is able to see besides people who are with you every day. Maybe needs help…just a thought, could or you couldn't.”
	“I got diabetes and I was not able to take care of myself all alone.”
Caregiver financial constraints/employment status	“Had to give him formula and formula is pretty expensive…diapers, formula, wipes…those are essential for the baby…how am I going to afford this? No job, nothing…Even his clothes, I didn't have money to get it. My friends just gifted me that's how I had it.”
	“In the beginning, when I first started having children, I didn't think it affected them especially like if I wasn't working….they would never know…especially when they were younger. I think now that they are older, I know it affects them”
	“Working was like impossible.”
Household food insecurity	“In the beginning, when I first started having children, I didn't think it affected them especially like if…low on food…they would never know…especially when they were younger. I think now that they are older, I know it affects them”
Social support available to caregiver	“…what happened to me, my kids, they helped me out. His dad left me even when pregnant, so when he left me I was jobless and that moment in time and I just have my mom who is 65 years old so who is going to take care of both of us…Father, he left. I don't have any contact…he just gone away. Things were like pretty hard.”
	“Even his clothes, I didn't have money to get it. My friends just gifted me that's how I had it.”

#### (xiii) Recommendation

Caregivers overwhelmingly responded that that they would recommend the screening program. Reasons for recommending the program included: providing help to parents, providing a safe space where expression is encouraged and prioritized, the convenience of locating the program where caregivers already visit.

*“Yes, because to get the help to the parent and because you can feel like you can express how you feel, how you are being treated somewhere where you attend and the help that you can and cannot get via resources…*” “*Yes, I'll say I recommend because there are some women who don't want to express what they are feeling or how things are with them so this definitely encouraged like you have someone to explain to and things will be good…*”

## Discussion

In summary, 3 major themes—structure, process, and outcomes—and 13 subthemes were identified from this study of pediatric caregivers engaged in SHAPE SDH screening and referral program during well-child visits. Overall, caregivers were willing to recommend the SHAPE program, as they perceived that the program offered social support—an important outcome of SHAPE screening and intervention. Additionally, the use of trained patient navigators appeared to be instrumental to the successful implementation of the program in clinics, for navigators could provide patients and caregivers with the appropriate time and personal attention they needed to complete the survey and discuss their needs. Patient navigators also enabled timely coordination of referrals to community-based organizations and follow ups. With this in mind, providers seeking to create successful SDH programs would do well to well to employ trained navigators and integrate them into screening, referral, and follow-up phases of the program.

Despite overall satisfaction with the referral program, caregivers were less satisfied with the legal services referral process and outcomes. They cited lack of timely contact from the legal resource team and prematurity of the legal resource provided, which rendered the resource inappropriate. Also of note, was a discrepancy between caregiver expectations of legal referrals and actual referrals. This might be remedied by setting clearer, more accurate expectations of the capabilities of legal services and referral workflow prior to caregiver contact with the legal team.

Previous studies on SDH screening studies have already shown that SDH screening and referral programs can lead to increased use of referral resources for pediatric caregivers [23]. This study enhances our understanding of patient experiences and concerns via qualitative feedback and perspectives obtained directly from caregivers who participated in one such program. Moreover, this study brings the voice of caregivers to the forefront of program evaluation and can encourage future work to proceed with a patient-centered model of care.

One of the notable discussions was caregivers’ linking social determinants of health to their children’s health. At the start of the discussion, participants considered social determinants to be a separate entity from children’s health. When citing factors that could influence child health, parents initially suggested a number of direct determinants, such as exposures to particular illnesses or harm. However, with more discussion, caregivers began linking social determinants, such as financial constraints and social support, to the well-being and health of the child. Specifically, patients were able to understand how a child may internalize the stress that caregivers face when trying to provide with resource constraints. Additionally, patients recognized that a caregiver struggling with meeting socioeconomic needs (e.g. employment lack of childcare) or struggling with physical or mental illness will not have the resources to support dependent children. Once patients were able to link social determinants with health outcomes, it became clearer why the screening program could potentially improve child health outcomes.

This study was limited by the homogeneous and a small number of caregivers with respect to the length of time spent in the program. As a result, inferences from this study were limited to the socioeconomic and cultural context of this sample. Although caregivers had received screening, intake, and referrals, none had completed up to 12-weeks of follow up post-screening. Out of convenience and relative difficulty of engaging caregivers who completed the 12-week follow-up period, we included caregivers who were yet to complete all 12 weeks of post-screening follow-up. Inability to capture caregivers outside of the 12-week post-screening window served as a potential limitation to the inferences made by this study. Including caregivers who spent more time with the program could allow researchers to understand the perspectives of and outcomes for caregivers with protracted case resolution timelines, e.g. housing and/or immigration cases requiring legal intervention. Other limitations were patient navigator responses during the forum. Those seeking to replicate the program might wish to extend the post-screening follow up window in order to capture patients with protracted case resolution timelines. Although navigators brought up relevant issues, they could introduce biases and interfere with the collection of usable quotes by caregivers in attendance. Navigator statements at the patient forum, though revelatory, were subsequently excluded from this paper.

## Conclusion

Overall, the SHAPE SDH screening and referral program offered social support to caregivers. Caregivers reported appreciation of having appropriate time, convenient location and personal attention they needed to complete the survey and discussed their needs. They found patient navigators to be an essential component in the successful delivery of the program. We also identified one major area of improvement—legal service referral process—which tended to include more complex situations, required more transparency and better communication. As the prevalence of SDH screening and referral programs embedded in clinical settings grows, it is of paramount importance to conduct qualitative evaluations of the caregiver/patient experience.
